# Antiglycating Effect of Phenolics from the Chilean Currant *Ribes cucullatum* under Thermal Treatment

**DOI:** 10.3390/antiox10050665

**Published:** 2021-04-25

**Authors:** Felipe Ávila, Natalia Ravello, Camila Manriquez, Felipe Jiménez-Aspee, Guillermo Schmeda-Hirschmann, Cristina Theoduloz

**Affiliations:** 1 Escuela de Nutrición y Dietética, Facultad de Ciencias de la Salud, Campus Lircay, Universidad de Talca, Talca 3460000, Chile; mravello@utalca.cl (N.R.); cmanriquez14@alumnos.utalca.cl (C.M.); 2 Department of Food Biofunctionality, Institute of Nutritional Sciences, University of Hohenheim, 70599 Stuttgart, Germany; felipe.jimenez@nutres.de; 3 Laboratorio de Química de Productos Naturales, Instituto de Química de Recursos Naturales, Campus Lircay, Universidad de Talca, Talca 3460000, Chile; schmeda@utalca.cl; 4 Laboratorio de Cultivo Celular, Facultad de Ciencias de la Salud, Campus Lircay, Universidad de Talca, Talca 3460000, Chile; ctheodul@utalca.cl

**Keywords:** Chilean native berries, phenolic compounds, advanced glycation end products, glyoxal, protein crosslinking, quinoproteins

## Abstract

Numerous dietary polyphenols possess antiglicating activity, but the effects of thermal treatment on this activity are mostly unknown. The effect of thermal treatment in the antiglycating activity of polyphenolic enriched extracts (PEEs) from *Ribes cucullatum* towards glyoxal-induced glycation of sarcoplasmic proteins was assessed. Sarcoplasmic proteins from chicken, beef, salmon, and turkey, were incubated 2 h at 60 °C with and without glyoxal and different concentrations of PEEs (0.25, 0.5, 1, and 5 mg/mL). The antiglycating activity was evaluated by: (1) Lys and Arg consumption, (2) Carboxymethyl lysine (CML) generation, and (3) lipid-derived electrophiles inhibition in a gastric digestion model. Protective effects were observed against CML generation in proteins and a decrease of electrophiles in the gastric digestion model. A dose-dependent consumption of Lys and Arg in proteins/PEEs samples, indicated the possible occurrence of quinoproteins generation from the phenolics. Protein/PEEs incubations were assessed by: (1) High pressure liquid chromatography analysis, (2) Gel electrophoresis (SDS-PAGE), and (3) Redox cycling staining of quinoproteins. Protein/PEEs incubations produced: (1) Decrease in phenolics, (2) increase of protein crosslinking, and (3) dose-dependent generation of quinoproteins. We demonstrate that phenolic compounds from *R. cucullatum* under thermal treatment act as antiglycating agents, but oxidative reactions occurs at high concentrations, generating protein crosslinking and quinoproteins.

## 1. Introduction

Thermal processing of food involves temperatures from 50 to 150 °C [1] and plays a fundamental role in food safety through the removal of bacteria and toxins, including lectins and protease inhibitors [2]. However, this process leads to the occurrence of numerous non-enzymatic chemical modifications in proteins, changing their chemical and nutritional properties [3]. One of the main chemical modifications occurring in proteins during thermal processing of foods is the Maillard reaction, which generates numerous chemical entities collectively denominated as advanced glycation end products (AGEs) [3]. The accumulation of AGEs has been related with numerous non-communicable chronic diseases (NCD), including diabetes, Alzheimer’s disease, and nuclear cataract [4,5]. AGEs are produced by non-enzymatic reactions occurring between nucleophilic groups arising from amino acids, proteins, lipids, and nucleic acids with carbonyl groups from reducing sugars or its oxidation products [3]. In proteins, the most susceptible amino acids to undergo Maillard reaction are N-terminal amino acids and side-chains of Cys, Lys, Arg, and His residues [3]. This reaction can occur under physiological conditions, but also during the thermal processing of foods [6]. The diet is an important contributor to the total pool of endogenous AGEs [7]. Moreover, it has been reported that a long term reduction of dietary AGEs can have beneficial effects in terms of cardio metabolic parameters such as a reduction of total cholesterol and low density lipoproteins (LDL) levels [8]. A decrease in markers of insulin resistance, oxidative stress and inflammation has also been reported [8,9]. Therefore, the development of AGEs inhibitors, produced both physiologically or during thermal processing of food, can have beneficial effects in the reduction of NCD. Aminoguanidine has been widely used as AGEs inhibitor. This molecule has two key nucleophilic reaction centers, namely: The hydrazine and the guanidine groups. Both groups can react with electrophilic molecules, including dicarbonyl compounds, to generate 3-amino-1,2,4-triazine derivatives [10]. However, due to toxicological concern its therapeutic use has been limited [10].

Numerous isolated and plant-derived phenolic compounds have shown antiglycating activity under physiological conditions (37 °C, pH 7.4) [11,12]. For example, the antiglycating activity of Canadian native berries and tropical medicinal herbs correlates strongly with the free radical scavenging activity and the total phenolic content [13,14]. However, the antiglycating effect of phenolic compounds under thermal processing has been less studied.

The intake of ground burgers prepared with a high polyphenolic content from red wine, a spice mixture or a mixture of berries, have shown to reduce the levels of malondialdehyde (MDA) in human plasma and/or urine [15,16,17]. These effects could be attributed to a decrease in lipid peroxidation occurring in the stomach during digestion as well as during cooking [15,16,17]. Lipid peroxidation is a well-known pathway to generate electrophiles such as MDA or glyoxal, which can react with free amino groups or proteins to generate advanced lipoperoxidation end products (ALEs) and AGEs [18]. Therefore, berries could be a good source of inhibitors of AGEs or ALEs by electrophile scavenging reactions, among others.

Chilean native berries have been widely assessed for their high antioxidant capacity, which correlates with their high polyphenolic content [19]. *Ribes cucullatum* (Grossulariaceae) is an endemic currant from the southern part of South America (Chile and Argentina) and the chemical composition of polyphenol-enriched extracts (PEEs) from *R. cucullatum* has been described by our group [20]. PEEs from *R. cucullatum* have shown high antioxidant capacity (determined by means of ORAC and superoxide radical anion scavenging assays), when compared with *R. magellanicum* or *R. punctatum* [21]. The analysis of the main phenolic constituents showed high levels of anthocyanins and hydroxycinnamic acids, namely: Cyanidin 3-rutinoside, cyanidin-3-glucoside, delphinidin-3-rutinoside, delphinidin-3-glucoside, 3-caffeoylquinic acid and 3-p-coumaroylquinic acid [21]. However, the antiglycating capacity of PEEs from *R. cucullatum* has not been assessed. Moreover, there is a lack of knowledge about whether phenolics could still exert antiglycating capacity under thermal treatment, considering the numerous chemical reactions that can take place under this condition.

In the present work, the antiglycating effect of the PEEs from *R. cucullatum* under thermal treatment conditions was assessed. We show that besides the antiglycating effect, thermal treatment of polyphenols can also induce chemical modifications in proteins generating protein crosslinking and quinoproteins.

## 2. Materials and Methods

### 2.1. Chemicals

From Sigma-Aldrich (St. Louis, MO, USA): NaHPO_4_·7H_2_O, NaH_2_PO_4_·2H_2_O, NaOH, 6-aminocaproic acid, L-Arginine, Nitroblue tetrazolium, Ethilenediaminetetraacetic acid (EDTA), glyoxal, Tween 20, fluorescamine, NaOH, HCl, trichloroacetic acid, glycine, thiobarbituric acid and aminoguanidine hydrochloride. 9,10-Phenanthrenequinone was purchased from Merck (Darmstadt, Germany). Ultrapure water was obtained from a Barnstead EasyPure water filter system (Thermo Scientific, Dubuque, IA, USA) and it was used for the preparation of all solutions.

### 2.2. Fruit Collection and Polyphenol-Enriched Extract (PEE) Preparation

Ripe fruits from *Ribes cucullatum* were collected in January 2015 in the Parque Nacional Conguillio, Region de la Araucanía, Chile [21]. Samples were kept at 4 °C until arrival to the laboratory. Voucher herbarium specimens were kept at the Herbario of the Universidad de Talca.

Briefly, fresh fruits were washed and homogenized in a blender with 50 mL of water. The mixture was extracted with 4 L of MeOH-formic acid (99:1 *v*/*v*) and extracted for 15 min in a sonicator bath (35 KHz, Elma Transonic 700, Elma GmbH & Co. KG, Singen, Germany). The extraction procedure was repeated three times. The extract was concentrated under reduced pressure. Distilled water was added and the resulting solution was enriched in polyphenols by means of an Amberlite XAD-7 HP resin (Sigma-Aldrich, St. Louis, MO, USA). The column was rinsed with distilled water and polyphenols were eluted with MeOH-formic acid (99:1, *v*/*v*). The eluate was evaporated to dryness and then lyophilized under reduced pressure to yield the polyphenol-enriched extract (PEE) [21].

The copigments and anthocyanin fractions were obtained from PEE from *R. cucullatum* by adsorptive membrane chromatography [20]. Briefly, PEE was dissolved in acidified MeOH (acetic acid 5% *v*/*v* in MeOH) and filtered before to be separated by the adsorption membrane. A preconditioned Sartobind S IEX 150 mL (Sartorius Stedim Biotech, Göttingen, Germany) membrane was used. The copigment fraction was collected in acidified MeOH (acetic acid 5% *v*/*v* in MeOH) and volatile compounds were removed by reduced pressure and the remaining solvent was freeze-dried. Anthocyanin fraction was eluted with NaCl 2.9% *w*/*v* in MeOH. NaCl was removed by means of Amberlite XAD7HP (Sigma-Aldrich, St. Louis, MO, USA) purification. The solvent was removed by reduced pressure evaporation and posterior freeze-dried.

### 2.3. Sarcoplasmic Protein Extraction

Water-soluble sarcoplasmic proteins were extracted according to Eady et al. [22] with minor modifications. Briefly, deboned samples of pectoralis major muscle from chicken and turkey, as well as bovine longissimus dorsi muscle and lateral muscle from salmon were obtained from a commercial packing through a local meat supplier. The medial portion of meat sample from each species analyzed (except salmon) was removed and trimmed of fat and connective tissue. Approximately 100 g of sample was homogenized in a Potter homogenizer at 35,000 rpm for 120 s, at 4 °C, with 20 mM phosphate buffer pH 6.5. The suspension was centrifuged at 7000× *g* at 4 °C during 15 min. The supernatant fraction containing the sarcoplasmic proteins was stored at −20 °C until use (1 to 32 weeks).

Sarcoplasmic proteins from salmon were extracted according to Nazar et al. [23]. Muscle was thawed and homogenized in 10 mM phosphate buffer, 0.15 M KCl and 0.1 mM EDTA, final pH 6.5. The homogenate was centrifuged during 15 min at 7000× *g*. All procedures were performed at 4 °C. The supernatant fraction containing the sarcoplasmic proteins was stored at −20 °C until use (1 to 32 weeks). Protein concentration was determined by means of the PierceTM BCA Protein Assay Kit (Thermo Scientific, Rockford, IL, USA), according to the manufacturer’s instructions.

### 2.4. Incubation of Sarcoplasmic Proteins with Glyoxal and PEE from R. cucullatum

Sarcoplasmic proteins were incubated at a final concentration of 3 mg/mL in presence and absence of glyoxal 10 mM and the PEE from *R. cucullatum* (0.25, 0.5, 1, and 5 mg/mL). Samples and glyoxal were prepared in phosphate buffer 100 mM at pH 7.4. The samples were incubated during 2 h at 60 °C. Controls in the absence of glyoxal and PEE were incubated at 4 and 60 °C during 2 h. At the end of the incubation, proteins were exhaustively dialyzed against phosphate buffer 100 mM, pH 7.4.

### 2.5. Quantification of Lysine Residues in Proteins Exposed to Glyoxal and PEE from R. cucullatum

Lysine residues in proteins were quantified by means of the fluorescamine method with minor modifications [24]. Proteins were adjusted to a concentration of 3 mg/mL and 0.05 mg of protein was added to 1500 μL of borate buffer (0.1 M, pH 9) together with 500 μL of fluorescamine (0.3 mg/mL). Samples were vortexed and incubated at room temperature (25 °C) during 10 min in the dark. Fluorescence was measured in a spectrofluorimeter (LS 55, Perkin Elmer, Llantrisant, UK) setting the excitation wavelength (λEX) at 390 nm and the emission wavelength (λEM) at 475 nm. Relative fluorescence was interpolated in a calibration curve prepared with 6-aminocaproic acid (concentrations ranging from 0 to 250 μM) and expressed as a %. The 100% was the amount of Lys present in proteins incubated during two hours at 60 °C.

### 2.6. Quantification of Arginine Residues in Proteins Exposed to Glyoxal and PEE from R. cucullatum

The Arg content present in sarcoplasmic proteins exposed to glyoxal and PEEs were quantified by means the 9,10-phenanthrenequinone method, with minor modifications [24]. Sarcoplasmic proteins at 3 mg/mL were diluted to 0.5 mg/mL with phosphate buffer 100 mM (pH 7.4) and 75 μg of the proteins were added to 300 μL of NaOH 1 M. Then, 900 μL of 9,10-phenanthrenequinone were added to the mixture and samples were homogenized and incubated during 1 h at 60 °C. The reaction was stopped by the addition of 1350 μL HCl (1.2 M). Fluorescence was measured in a spectrofluorimeter setting λEX at 312 nm and λEM at 392 nm. Relative fluorescence was interpolated in a calibration curve prepared with L-Arg as standard (concentrations ranging from 0 to 100 μM), and expressed as a %. The amount of Arg present in proteins incubated during 2 h at 60 °C was considered as the 100%.

### 2.7. Sodium Dodecyl Sulfate Polyacrylamide Gel Electrophoresis (SDS-PAGE) Analysis

Irreversible protein crosslinking was determined by means of SDS-PAGE analysis under reducing conditions. The samples 22.5 μg of protein per sample in Tris buffer (62.5 mM, pH 6.8), containing 2% SDS, 10% glycerol, 100 mM β-mercaptoethanol, and bromophenol blue as a tracking dye, were boiled for 5 min. Stacking and resolving gels were prepared at 3% (*w*/*v*) and 12% (*w*/*v*) acrylamide, respectively. A running buffer composed by 25 mM Tris, 400 mM Gly and 0.1 % SDS, pH 8.3 was used. Electrophoresis was carried out at 100 V for 60 min. Afterwards, gels were stained with 0.1% Coomassie Brilliant Blue and distained in solution of 10 % (*v*/*v*) ethanol and 0.75 % (*v*/*v*) acetic acid up to 24 h. Gels were scanned and the quantification of the crosslinked protein was performed with Image J software (NIH, Bethesda, MD, USA). Protein crosslinking was quantified considering the proteins with molecular masses higher than ~50 kDa.

### 2.8. Immunochemical Detection and Quantification of Carboxymethyl-Lysine (CML)

Gels were electrotransfered (100 V, 60 min) onto a Hybond nitrocellulose membrane (GE Healthcare, Saclay, France). Afterwards, the membrane was incubated with blocking buffer containing phosphate buffered saline (PBS) with 1% bovine serum albumin (BSA) and Tween 20 (0.1%) during 2 h at room temperature. Blocked-membranes were then incubated overnight under agitation at 4 °C with a primary monoclonal antibody anti-carboxymethyl-lysine (R&D Systems, Minneapolis, MN, USA) using a dilution of 1/1000 in blocking buffer. Then membranes were washed with PBS, and incubated with horseradish peroxidase-conjugated secondary antibodies. Membranes were revealed with ECL Plus chemiluminescent detection system (Amersham, GE Healthcare, Buckinghamshire, UK). Blots were scanned and signal intensities were quantified with ImageJ software (1.48v, NIH, Bethesda, MD, USA).

### 2.9. Oxygen Consumption Analyses

The PEE from *R. cucullatum* (5 mg/mL) was dissolved in phosphate buffer 100 mM, pH 7.4 and incubated in a sealed vial at 60 °C for 2 h at dark. Dissolved oxygen amounts were carried out using of a Clark-type polarographic probe (Hanna Instruments, HI5421, Woonsocket, RI, USA), calibrated at two points (0.00 mg /L and 8.26 mg/L). Dissolved oxygen measurements were determined initially (time 0) and after 2 h of incubation. Before the measurements, samples were incubated to reach constant temperatures of 25 °C. Catalase (50 μg/mL) was added for samples incubated for 2 h at 60 °C at the end of the oxygen measurements.

### 2.10. Identification and Quantification of Main Phenolic Compounds Present in the PEE from R. cucullatum Exposed to Thermal Processing

Phenolic compounds present in PEEs, co-pigments and anthocyanin fractions were analyzed by means of high pressure liquid chromatography using diode-array detector (HPLC-DAD) according to chromatographic conditions previously described [20]. Phenolic compounds identification was based on retention time, UV spectra and mass spectrometry data, as previously described by Jimenez Aspee et al. [20].

The PEE from *R. cucullatum* (5 mg/mL) was dissolved in phosphate buffer 100 mM, pH 7.4, bubbled during 20 min with 20% O_2_ and incubated in the presence or absence of sarcoplasmic proteins (3 mg/mL) from chicken in the dark and at 4 °C and 60 °C. Then, samples were deproteinized by means of ultrafiltration, using a PierceTM Protein concentrator, PES, 3MWCO, according to the manufacturer’s instructions (Thermo Fisher Scientific, Rockford, IL, USA). Samples were analyzed by means of HPLC-DAD in a Shimadzu Prominence device (Shimadzu Co., Kyoto, Japan), equipped with an LC-20AT pump, SPD-M20A UV diode array detector and CTO-20AC column oven, and Labsolution software (Shimadzu Co., Kyoto, Japan) The analyses were done using a MultoHigh 100 C18 column, 5 µm, 250 × 4.6 mm (CS-Chromatographie Service Gmbh., Langerwehe, Germany). The column was maintained at 30 °C and a linear gradient solvent system was used consisting of H_2_O-formic acid-acetonitrile (87:5:3, *v*/*v*/*v*, solvent A) and H_2_O-formic acid-acetonitrile (40:5:50, *v*/*v*/*v*, solvent B).

Quantification of anthocyanins and chlorogenic acid was carried out using external six-point calibration curves built with the commercial standards: Delphinidin 3-glucoside (98.0% purity, 89,627), cyanidin 3-glucoside (99.3% purity, 89,616), and cholorgenic acid (98.9% purity, 89,175) The samples were injected three times and peak areas were determined at 520 nm and 320 nm for anthocyanins and chlorogenic acid, respectively. The peak areas were interpolated in a calibration curve and results were expressed as μg/g of sample.

### 2.11. Protein-Quinone Conjugation Determination by Means of Redox Cycling Staining

Protein-quinone adducts were determined according to the method of Paz, Fluckiger, Boak, Kagan, and Gallop (1991). Sarcoplasmic proteins from turkey (pectoralis major muscle) or bovine (longissimus dorsi muscle) (3 mg/mL) were incubated with the PEE from R. cucullatum at different concentrations (5, 1, 0.5, 0.25 mg/mL), in the absence of glyoxal. Controls corresponding to proteins incubated with or without glyoxal (10 mg/mL) were incubated during 2 h at 60 °C. Additionally, a control of the proteins unmodified was incubated during 2 h at 4 °C, in the absence of glyoxal. Proteins were separated by means of SDS-PAGE under non-reducing conditions and then electro-transferred onto a nitrocellulose membrane according to the procedure described above. After transference, the membrane was stained with a solution containing Nitroblue tetrazolium (0.24 mM) in glycine (2 M), pH 10.

### 2.12. Determination of Lipid-Derived Electrophiles Content after Simulated Gastric Digestion

The content of lipid-derived electrophiles, expressed as malondialdehyde (MDA) content, after simulated gastric digestion was determined according to Kanner, Selhub, Shpaizer, Rabkin, Shacham, and Tirosh (2017), with minor modifications. The simulated gastric fluid (SGF) contained NaCl (0.2% *w*/*v*), pepsin (0.32% *w*/*v*) and 700 µL of HCl (37%) in a final volume of 100 mL of ultrapure water. Thirty grams of turkey meat were ground with 90 mL of the SGF solution for 1 min in a Waring blender (Thomas TH-501V, Thomas Elektrogeräte, Shanghai, China). The pH of this solution was adjusted to 3.0 with HCl 7 N. The PEE from *R. cucullatum* (0–500 µg/mL) was incubated with the meat-liquid mixture in a shaking water bath at 37 °C during 60 min. Afterwards, 800 µL of the samples, controls and blanks were centrifuged at 20,000× *g* for 15 min at 4 °C. The MDA content was determined by means of the thiobarbituric acid (TBA) in the supernatant resulting from the centrifugation (750 µL), which was mixed with 750 µL of TBA solution (0.37% TBA, 15% trichloroacetic acid in 0.25 N HCl), and boiled during 20 min. Absorbances were determined at 532 nm. Results are expressed as IC50 values (μg/mL). Aminoguanidine was used as a standard inhibitor.

### 2.13. Statistical Analyses

Experiments were performed in triplicate and results were expressed as mean values ± SD. Statistical differences between the means of different treatments were determined by one way analysis of variance (ANOVA) followed by Dunnett’s (Lys and Arg quantification) or Tukey’s post-hoc (MDA and phenolic compound analyses) test. Statistical analyses were performed with the software SPSS 14.0 for Windows (IBM, Armonk, NY, USA), significance was set at *p* < 0.05.

## 3. Results

### 3.1. Characterization of Phenolic Compounds from Ribes cucullatum by Means of HPLC-DAD Analyses

**Figure 1:**
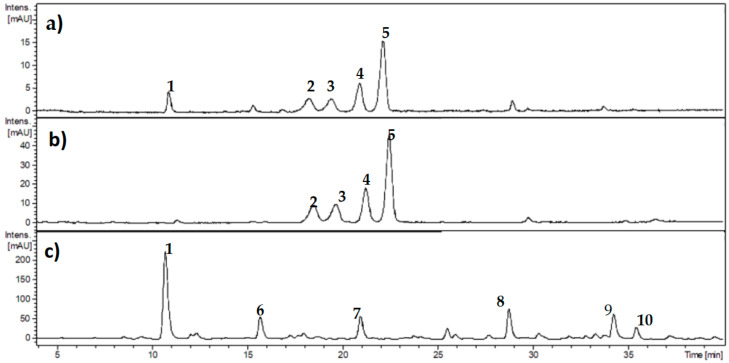
Representative chromatograms obtained from high-performance liquid chromatography analysis of *Ribes cucullatum* extracts detecting the absorbance at 280 nm. Panel (**a**) shows analysis of polyphenol enriched-extract from *Ribes cucullatum* berries; panel (**b**) shows analysis of anthocyanin fraction; and panel (**c**) shows analysis of copigments fraction. Numbers indicate tentative identification: 1: 3-caffeoylquinic acid (chlorogenic acid); 2: delphinidin-3-O-rutinoside; 3: delphinidin-3-*O*-glucoside; 4: cyanidin-3-*O*-rutinoside; 5: cyanidin-3-*O*-glucoside; 6: 3-p-coumaroylquinic acid; 7: Caffeoylquinic acid isomer; 8: 3-feruloylquinic acid; 9: Kaempeferol-3-*O*-rutinoside; 10: Kaempferol-3-*O*-glucoside.

Phenolic composition of *Ribes cucullatum* was analyzed by means of HPLC-DAD. Figure 1a,b show the detection of 5 main peaks from the analysis of PEEs. Four of them were also detected in the anthocyanin enriched fraction (Figure 1b), with the exception of chlorogenic acid. Figure 1c depicts a chromatogram from the co-pigments fraction, showing non-anthocyanin phenolics tentatively identified as: 3-caffeoylquinic acid (peak number 1); 3-p-coumaroylquinic acid (peak number 6); Caffeoylquinic acid isomer (peak number 7); 3-feruloylquinic acid (peak number 8); Kaempeferol-3-O-rutinoside (peak number 9) and Kaempferol-3-*O*-glucoside (peak number 10).

### 3.2. Evaluation of Antiglycating Activity of the PEE from R. cucullatum

The effect of thermal processing of *R. cucullatum* to decrease lipid peroxidation electrophiles, in a model relevant for endogenous accumulation of AGEs and ALEs, was assessed by means of in vitro gastric digestion of turkey meat (Table 1). The capacity of the PEE from *R. cucullatum* was expressed as IC_50_ of malondialdehyde (MDA) content. The results were compared with PEEs exposed to 60 °C during 2 h. IC_50_ values were also obtained for anthocyanin and copigment fractions without heating and compared with the aminoguanidine (AG) control. Table 1 shows that the lowest IC_50_ value was obtained for the PEE from *R. cucullatum* without heating. The PEE was approximately 5 times more efficient to decrease the generation of MDA when compared to AG. It can also be observed that the anthocyanin fraction was also less effective to inhibit the MDA generation. The exposition of the PEE to the thermal treatment induced a significant increase in the MDA content, when compared with non-thermally treated samples (Table 1).

The antiglycating effect of the PEE (the most efficient fraction to decrease electrophiles arising from gastric digestion) from *R. cucullatum* was evaluated quantifying the amount of Lys and Arg residues present in sarcoplasmic proteins from turkey, chicken, beef and salmon meats. The experimental treatments included the following conditions: (1) Sarcoplasmic proteins incubated at 4 °C during 2 h; (2) sarcoplasmic proteins incubated at 60 °C during 2 h; (3) sarcoplasmic proteins incubated with different concentrations of the PEE from *R. cucullatum* at 60 °C during 2 h; (4) sarcoplasmic proteins incubated with glyoxal at 60 °C during 2 h; (5) sarcoplasmic proteins incubated with glyoxal and PEE from *R. cucullatum* at 60 °C during 2 h; and (6) sarcoplasmic proteins incubated with glyoxal and aminoguanidine at 60 °C during 2 h. The concentration of glyoxal (10 mM) was selected experimentally and corresponds to an amount where a statistically significant (*p* < 0.05) reduction of the amino acids Lys and Arg were observed for all kind of meats after incubation for 2 h at 60 °C. The concentrations of aminoguanidine (0.25; 0.5; 1; 5 mg/mL) were chosen to have a stoichiometric ratio aminoguanidine:glyoxal of 0.22:1; 0.45:1; 0.9:1; 4.5:1, respectively. Whereas, the concentrations of *R. cucullatum* were chosen in order to compare their protective effects with aminoguanidine. On the other hand, the molar ratio Lys:glyoxal were calculated to be 0.16:1 for chicken; 0.08:1 for bovine; 0.14:1 for salmon and 0.1:1 turkey meat. Therefore, protective effects against protein glycation could be expected from aminoguanidine and *R. cucullatum*.

**Figure 2:**
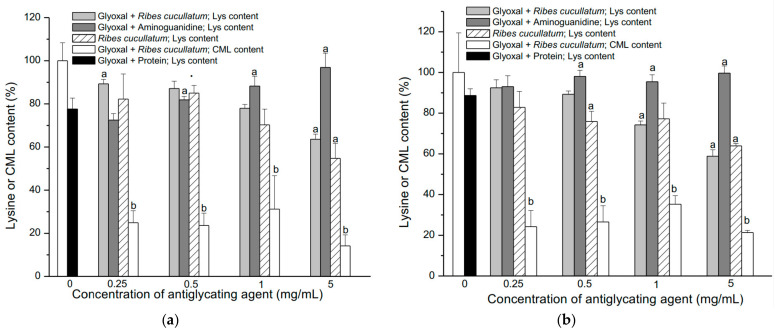

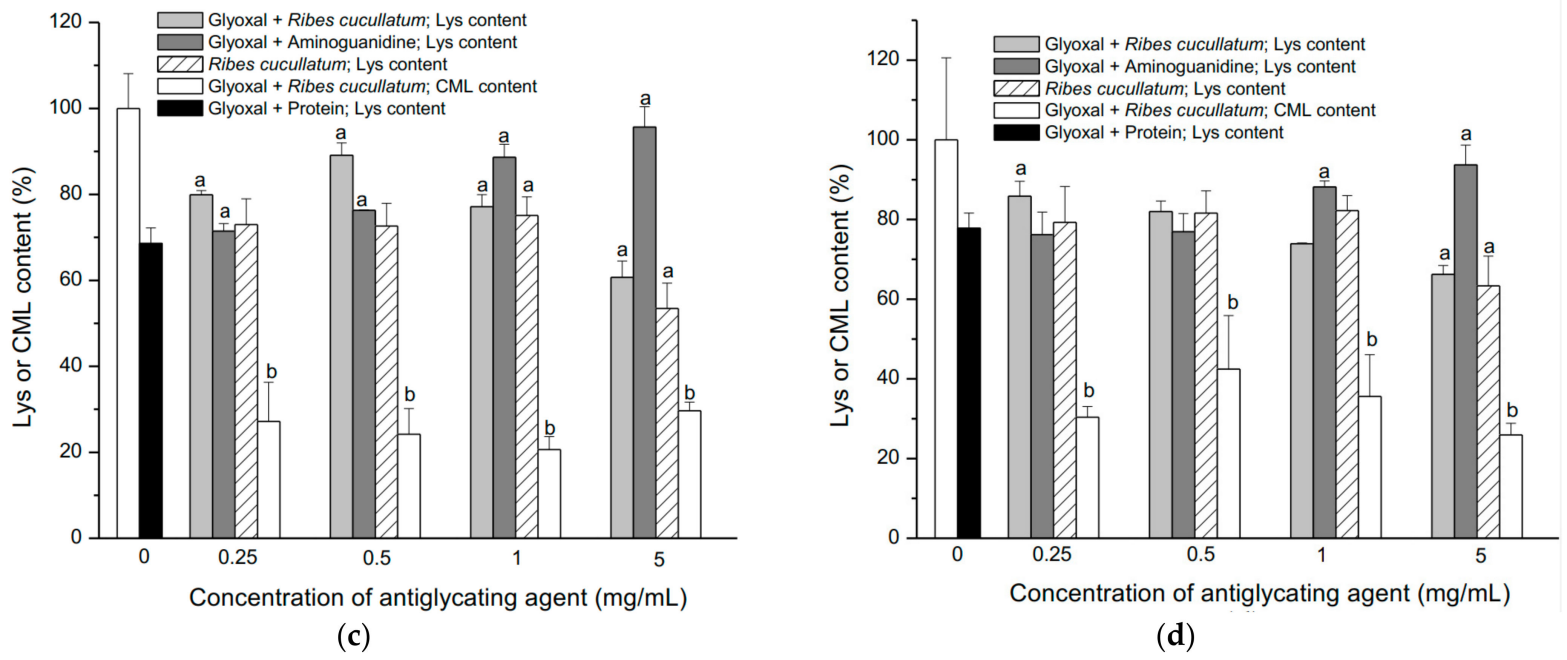
Effect of glycation induced by glyoxal (10 mM) on the amount of Lys residues and *N*-ε-carboxymethyl lysine (CML) levels in proteins of four different types of meat: Panel (**a**) Chicken; (**b**) Beef, (**c**) Salmon and (**d**) Turkey. The content of CML was normalized considered proteins glycated with glyoxal as 100 %. Letters a and b indicate significant differences (*p* < 0.05) when compared with proteins incubated with glyoxal for Lys (black column) or CML (white column) determinations, respectively.

Figure 2a–d (dashed line) shows that the Lys content was significantly reduced in sarcoplasmic proteins from all the meat types assessed. The salmon meat presented the highest Lys consumption (Figure 2c).

The antiglycating capacity of the PEE from *R. cucullatum* was also evaluated determining the levels of carboxymethyl lysine (CML) generation. Glyoxal can react with ε-amino groups from Lys in proteins to generate CML, which was significantly inhibited by PEEs from *R. cucullatum*, even at the lowest concentration (0.25 mg/mL), for all kind of meats (Figure 2a–d).

On the other hand, the lowest concentration of aminoguanidine (AG, 0.25 mg/mL) showed statistically significant protection only in salmon proteins (Figure 2c). A significant antiglycating effect of Lys residues was observed at AG concentrations over 0.5 mg/mL and the inhibition behavior tended to reach a plateau at the highest AG concentrations (Figure 2a–d). Significant antiglycating effects of Lys moieties in the mixture protein/glyoxal/PEE were observed for all the meat samples at the lowest concentration (Figure 2a–d). The control samples composed by protein/PEE showed a decrease in the Lys amount at higher doses of *R. cucullatum* for proteins from all the meat samples (Figure 2a–d). This behavior was the opposite of aminoguanidine, a widely used antiglycating compound.

**Figure 3:**
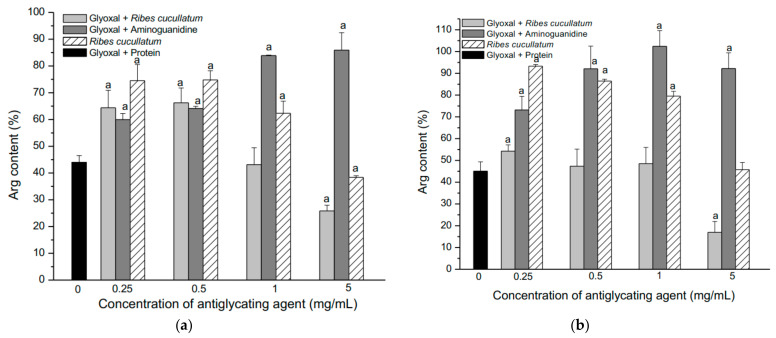

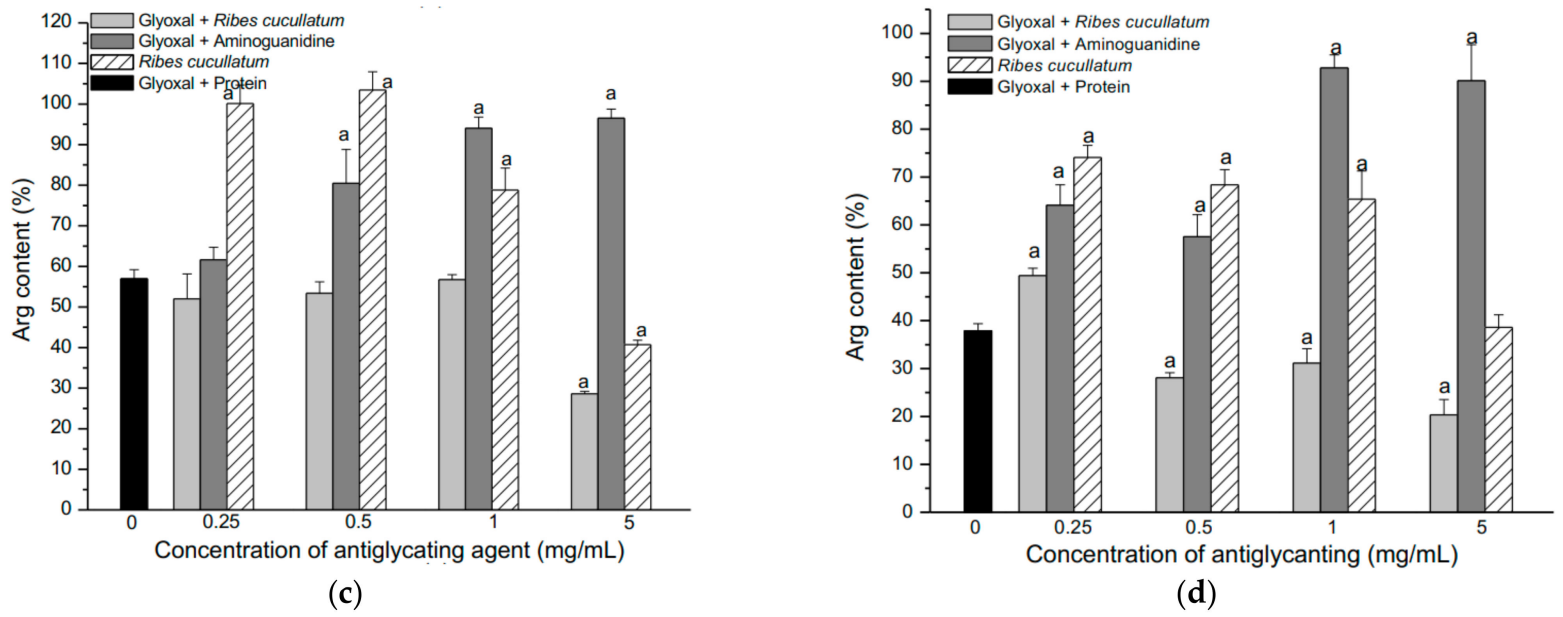
Effect of glycation induced by glyoxal (10 mM) on the concentration of Arg residues in proteins of four different types of meat: Panel (**a**) Chicken; (**b**) Beef; (**c**) Salmon and (**d**) Turkey. Letter a indicates significant differences (*p* < 0.05) when compared with proteins incubated with glyoxal (black column).

The effectiveness of aminoguanidine and the PEE from *R. cucullatum* to inhibit Arg glycation was also evaluated. Figure 3 shows the changes in the Arg content from the 4 different types of meats exposed to the treatments described above.

Glyoxal induced a significant decrease of Arg in all the studied meat types, showing that turkey meat had highest decrease (Figure 3a–d, dashed line). The AG showed a significant protection at the lowest concentration (0.25 mg/mL) for all meat types, except salmon meat (Figure 3a–d). On the other hand, AG at 0.5 mg/mL significantly protected against glyoxal in all the meat proteins studied (Figure 3a–d). The Arg content reached a plateau at higher AG concentrations, in the same way than the Lys consumption (Figure 2a–d). The antiglycating effects of the PEE from *R. cucullatum* were observed at 0.25 mg/mL in proteins from all meat types, except salmon meat (Figure 3a–d). In the mixture protein/glyoxal/PEE, at higher concentrations of *R. cucullatum* a decrease in the Arg content was observed (Figure 3a–d).

The same trend was observed in the analysis of the Lys levels in the mixture protein/glyoxal/PEE (Figure 2). Incubation of the mixtures protein/*R. cucullatum* showed also a decrease of Arg residues when increasing doses of *R. cucullatum* (Figure 3a–d).

### 3.3. Proteomic and HPLC Analyses of the Effects Induced by PEE from R. cucullatum under Thermal Degradation Conditions in Meat Proteins

SDS-PAGE was carried out to determine if the decrease in Lys and Arg induced by the PEE from *R. cucullatum* was associated with protein crosslinking. For this purpose, different concentrations of the mixture protein/PEE were incubated at 60 °C and compared with the mixture protein/glyoxal under the same conditions, as well as with proteins incubated at 4 and 60 °C.

**Figure 4:**
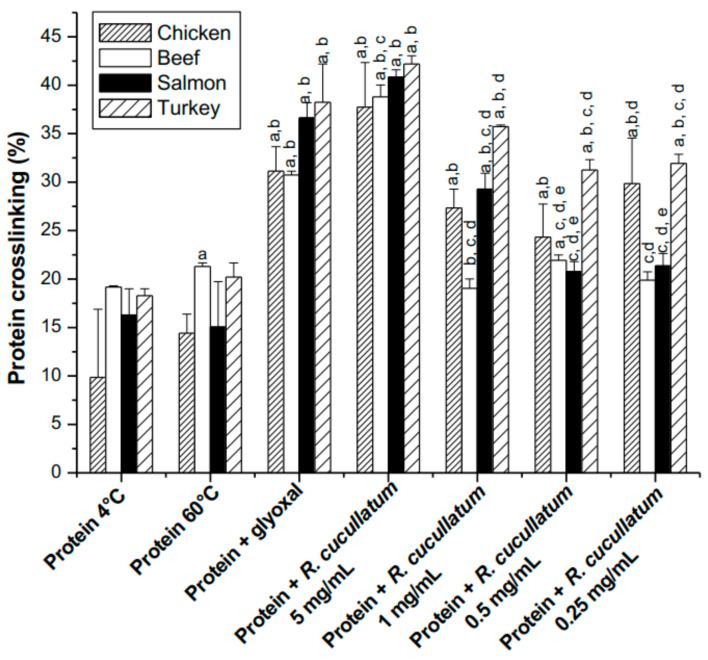
Densitometric analysis of 1D SDS-PAGE under reducing conditions, showing protein crosslinking (proteins molecular mass higher than ~50 kDa) from of sarcoplasmatic proteins from chicken, beef, salmon and turkey incubated with glyoxal (10 mM) or different concentrations of PEE from *R. cucullatum* at 60 °C during 2 h. Statistically significant differences (*p* < 0.05) between indicated samples and the different treatments, within the same kind of meat, is indicated with letters as follows: (a) Protein at 4 °C; (b) protein 60 °C; (c) protein glyoxal; (d) protein + *R. cuculltaum* 5 mg/mL; (e) protein + *R. cuculltaum* 1 mg/mL.

No significant protein crosslinking, except for beef proteins was observed, in the densitometric analyses of SDS-PAGE gels (under reducing conditions) when proteins were exposed to 60 °C during 2 h (Figure 4).

The positive control protein/glyoxal showed a significant increase in protein crosslinking (molecular mass >50 kDa) for all kind of meats. The area under the curve of protein crosslinking was calculated with different PEE concentrations. The trend for protein crosslinking induced by glyoxal indicates that the most susceptible species are turkey > chicken~salmon > beef (Figure 4). However, it should be noticed that this data could be affected by the generation of high molecular mass aggregates that do not enter into the gel (molecular mass >250 kDa), and consequently were not quantified in these analyses.

With the aim to determine the possibility of quinoproteins generation induced by phenolic compounds from *R. cucullatum*, HPLC-DAD quantification of main compounds was carried out to determine whether polyphenols from *R. cucullatum* could be oxidized under the different thermal conditions used. Table 1 shows that the content of anthocyanins decreased after the incubation (2 h at 60 °C). The highest reduction was observed for cyanidin-3-rutinoside, followed by cyanidin-3-glucoside (Table 2).

The HPLC-DAD quantification of main compounds was carried out to determine whether polyphenols from *R. cucullatum* could be oxidized under the different thermal conditions. Table 2 shows that the content of anthocyanins decreased after the incubation (2 h at 60 °C). The highest reduction was observed for cyanidin-3-rutinoside, followed by cyanidin-3-glucoside (Table 2). The HPLC analyses of the mixture resulting from the incubation of chicken sarcoplasmic proteins incubated with the PEE from *R. cucullatum* at 60 °C during 2 h showed a significant decrease of the five phenolic compounds quantified, compared with the mixtures incubated at 4 °C (Table 2). These results indicate that the addition of the protein reduces the amount of phenolic compounds of the PEE from *R. cucullatum*, which could be associated to a covalent or electrostatic attachment of the phenolic compounds to the protein.

**Figure 5:**
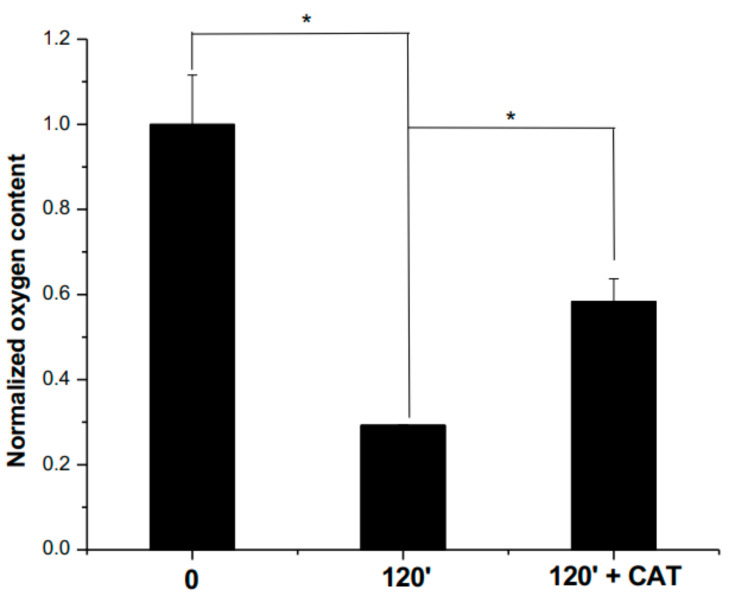
Normalized oxygen content present in *R. cucullatum* (5 mg/mL) solutions exposed during 120 min at 60 °C. CAT indicates the addition of catalase (50 μg/mL). * Indicates statistically significant differences (*p* < 0.05), compared to the indicated sample.

The occurrence of oxidative stress induced by the PEE from *R. cucullatum* during incubations at 60 °C was determined by O_2_ consumption analyses (Figure 5).

A decrease of 62.1 % in the O_2_ concentration after 2 h of incubation at 60 °C was observed when compared with samples at time 0 (Figure 5). The O_2_ concentration increased by 20.4% after the addition of catalase (50 μg/mL) when compared with samples incubated during 120 min in the absence of catalase (Figure 5). Incubations of buffer with catalase did not show a significant increase of dissolved O_2_ (data not shown).

**Figure 6:**
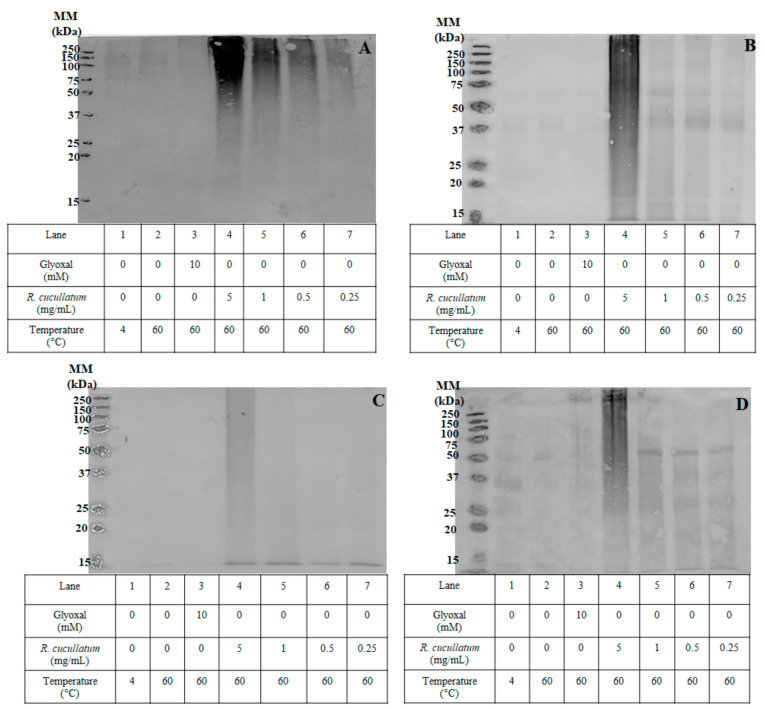
NBT-glycinate staining of quinoproteins generated by sarcoplasmatic proteins from: Panel (**A**) Chicken, (**B**) Beef, (**C**) Salmon, and (**D**) Turkey, incubated with glyoxal (10 mM) or different concentrations of PEE from *R. cucullatum* at 4 and 60 °C.

To determine the effect of thermal treatment on the capacity of PEEs from *R. cucullatum* to generate protein-bound quinones, redox-cycling staining was used for specific detection of quinoproteins. The nitroblue tetrazolium (NBT)-glycinate staining of quinoproteins is shown in Figure 6.

Proteins incubated at 4 °C or 60 °C or those incubated with glyoxal at 60 °C were not stained with NBT (Figure 6). On the other hand, when proteins from all the meat types were incubated with the PEE, staining was observed in a concentration–response manner. The stain was distributed along all the proteins with different molecular masses, being the high molecular mass proteins the most reactive ones (>50 kDa). This effect was especially evident when the PEE from *R. cucullatum* was evaluated at 5 mg/mL.

## 4. Discussion

Phenolic compounds derived from dietary intake are considered key molecules to prevent the onset of numerous age-related chronic diseases [25]. Our group has assessed previously the chemical composition and cytoprotective effects of the Patagonian currants *Ribes cucullatum, Ribes magellanicum, Ribes punctatum,* and *Ribes trilobum* [20]. We selected *R. cucullatum* to assess its antiglycating properties considering the PEEs yield and antioxidant capacity [20]. The anthocyanin composition of *R. cucullatum*, determined by liquid chromatography with electrospray ionization and mass spectrometry detection (LC-ESI/MS) analyses, identified the presence of 8 anthocyanins, with cyanidine and delphinidine (glucoside and rutinosides), as the main compounds [20], in agreement with our results (Figure 1, Table 2). On the other hand, 19 flavonoids and hydrocynamic acids were identified in the co-pigment fractions of *R. cucullatum* by means of LC-ESI/MS analyses [20], including 6 of the major compounds detected here by HPLC-DAD (Figure 1).

It has been demonstrated that when turkey meat is processed thermally with a high content of polyphenols from red wine, coffee or berries, the postprandial levels of plasmatic MDA in humans decrease, when compared to control meals [15,17,26]. It has been shown that phenolic compounds can scavenge electrophilic molecules such as MDA or dicarbonyl compounds, decreasing the amount of protein-derived adducts such as AGEs [27]. However, to date only few studies have evaluated simultaneously the antiglycating capacity of phenolic compounds under thermal processing conditions, as well as their effect on proteins (generation of crosslinking and quinoproteins). In this work, we have assessed the antiglycating properties of a PEE from *R. cucullatum* fruits on sarcoplasmic proteins from chicken, beef, salmon, and turkey. The antiglycating capacity of the PEE from *R. cucullatum* fruits was evaluated against glyoxal-induced glycation by complementary techniques, including: (1) Quantification of Lys and Arg amounts (2) determination of the levels of carboxymethyl lysine (CML) by immunochemistry and (3) determination of electrophilic compounds generated during gastric digestion of turkey meat. Glyoxal can react with ε-amino groups from Lys in proteins to generate CML, glyoxal-derived hydroimidazolone and glyoxal lysine dimer (GOLD). We have observed a significant inhibition of formation of CML in sarcoplasmic proteins exposed to glyoxal when incubations were performed in the presence of PEE from *R. cucullatum*. A decrease in the amount of CML and glyoxal by dietary polyphenols has been observed in the thermal processing of cookies [28]. In addition, scavenging reactions between methylglyoxal and quercetin have been reported to occur in the A-ring of the flavonoid [29]. We have observed that PEE from *R. cucullatum* exert a significant reduction of lipid-derived electrophiles levels (expressed as MDA IC_50_) generated by lipid peroxidation reactions in an in-vitro gastric digestion model. The IC_50_ values showed that PEE inhibits lipid peroxidation more efficiently than AF and CF fractions. This fact might be explained by synergistic effects mediated by phenolics from AF and CF as well as by the non-phenolic components of the PEE. Lipid peroxidation reactions generate different electrophilic compounds, including MDA, 4-hydroxynonenal, and glyoxal [18,30]. These compounds can react readily with proteins or amino acids to generate AGEs and ALEs [18]. The effects of PEEs from *Prumnoptis andina* and *Araucaria araucana* kernels on MDA levels, using the same model of gastric digestion, have been recently reported by our group [31,32]. Values of IC_50_ of 95.0 µg/mL were reported for *Prumnoptis andina* [31] and IC_50_ values ranged from 90.0 to 511.5 µg/mL for PEEs from *Araucaria araucana* [32], indicating that PEEs from *R.cucullatum* are more effective when compared to these species to decrease MDA levels, even after thermal treatment. Interestingly, we have found here a decrease in the effectiveness to inhibit MDA when PEE from *R. cucullatum* were exposed to thermal treatment. The inhibition of the electrophilic products arising from lipid peroxidation could proceed from multiple pathways, including scavenging of free radicals, hydroperoxides, and electrophilic compounds [33,34]. Therefore, the modulation of electrophilic compounds in the model of gastric digestion used in this work can also be related with the antioxidant activity, a relationship that has been previously established [13,14,35]. Although antiglycating activity has not been extensively assessed, the effects of thermal treatment on the antioxidant activity mediated by phenolic compounds in fruits and vegetables have been previously examined and divergent results have been obtained. It has been reported that total antioxidant activities of tomatoes (2, 15, and 30 min at 88 °C) [36], sweet corn (25 min, 115 °C) [37] and carrots (2 min, 70 °C, pressure 400–600 MPa for 15 min) [38] were enhanced with thermal processing, while antioxidant capacities of soybeans decreased with similar processes [39]. In the strawberry *Fragaria* x *ananassa*, the effect of thermal treatment (95 °C or 60 °C), in isolated anthocyanins or in the whole fruits, have resulted in a decrease of anthocyanins following a first order kinetics [40,41]. Interestingly, the thermal processing (40–80 °C) of maqui berry (*Aristotelia chilensis* [Mol] Stuntz) have proved to decrease its antioxidant capacity and phenolic content [42]. Also, an increase of free ellagic acid with the temperature, as a result of the conversion from hydrolysable tannins was observed [42].

We have observed a decrease in the levels of Lys and Arg when increasing concentrations of PEE in the mixture protein/glyoxal/PEEs, and for this two amino acids, the protective effects mediated by PEE against the consumption induced by glyoxal were only observed at the lowest concentration. The incubation of the mixture protein/PEEs also showed a dose-dependent decrease in the levels of Lys and Arg. This fact, together with the increase in the intermolecular protein crosslinking in the mixture protein/PEEs, suggests the occurrence of a nucleophile-electrophile interaction, in agreement with the consumption of the basic amino acids Lys and Arg [43]. The occurrence of protein crosslinking and the decrease in Lys and Arg residues by means of Maillard chemistry or Michael addition have been reported widely [43]. In addition, the occurrence of radical-radical reactions may also contribute to protein crosslinking [43]. The main amino acids involved in this kind of protein crosslinking are Cys, Trp, and Tyr [43]. However, a nucleophile-electrophile reaction occurring by His, Tyr, or Trp oxidation products containing carbonyl function and Lys or Arg can also generate protein crosslinking [43]. The oxygen consumption profiles indicate that oxidative reactions occur during thermal processing of the PEE from *R. cucullatum*. The increase in the amount of oxygen after the addition of catalase indicates the generation of H_2_O_2_ in our system, considering that catalase decompose H_2_O_2_ to O_2_ and H_2_O [44]. This approach has been used before to demonstrate the generation of reactive oxygen species [45,46]. Therefore, a contribution of free radical reactions to the protein crosslinking and oxidation cannot be discarded.

The consumption of Lys and Arg, as well as the increase in protein crosslinking found after the incubations of protein with PEE, could be mediated by polyphenol oxidation products. Consequently, a decrease in the amount of polyphenols should be expected when incubating protein with PEE. A reduction in the content of anthocyanins and chlorogenic acid was observed in the mixtures protein/PEE incubated at 60 °C. It has been shown that the degradation of cyanidin-3-glycoside occurs by at least two mechanisms: (1) Deglycosylation of the anthocyanin by excision of the sugar and posterior cleavage of the anthocyanidin, generating compounds such as 4-hydroxybenzoic acid or phoroglucinaldehyde [47]; and (2) oxidative pathways, which generate oxidation compounds including *O*-quinones [48]. The production of quinones in phenolic compounds including quercetin, epicatechin or chlorogenic acid has been previously reported [49]. The oxidation of cyanidin-3-glucoside and chlorogenic acid, catalyzed by polyphenol oxidases, also generates cyanidin 3-glucoside *O*-quinone [48]. This process could also occur with other 1,2-benzene diols, which might generate quinones [50]. The quinone moiety (two electron oxidation) can act as electrophile, reacting with nucleophilic residues in proteins such as Lys or Arg producing quinoproteins [51]. This quinoprotein could undergo a second Michael addition, producing inter or intra molecular crosslinking. This route could explain our results in terms of the generation of monomers and dimers, showing reactivity towards NBT. The reaction of quinones with proteins can generate protein crosslinking, as well as the consumption of the basic amino acids Lys and Arg [51]. It has been reported that the administration of 2-(glutathione-S-yl)-hydroquinone to rats generates quinoproteins, affecting mainly proteins with high contents of Lys residues [52]. On the other hand, the analysis of rate constants in the reaction between 4-methylbenzoquinone with Cys is 3 times higher compared with the reaction with N-α-acetyl-_L_-Lys [53].

High concentrations of PEE from *R. cucullatum* can react during thermal treatment with sarcoplasmic proteins, reducing Lys and Arg moieties with a concomitant generation of quinoproteins. This indicate that high concentrations of PEE from *R. cucullatum* might produce anti-nutritional effects, decreasing the levels of Lys, an essential amino acid. Therefore, concentrations of PEE from *R. cucullatum* should be carefully chosen to achieve antiglycating effects, but avoiding deleterious reactions with proteins during thermal processing of meats.

Despite the generation of quinoproteins, it is noteworthy that the IC_50_ of MDA generation for PEE from *R. cucullatum* is about 5 times lower than the IC_50_ of AG. Even though thermal processing of *R. cucullatum* decreases its effectiveness to inhibit the MDA generation, it is still 3.6 times more effective than AG. Therefore, phenolic compounds extracted from this native fruit are interesting to be investigated as a functional food.

## 5. Conclusions

Our results show that phenolic compounds from *R. cucullatum* can act as antiglycating agents during the thermal processing, decreasing CML levels in sarcoplasmic proteins and decreasing electrophiles generated by in-vitro gastric digestion of turkey meat. However, at high concentration PEEs from *R. cucullatum* can also produce oxidative modifications evidenced by O_2_ consumption experiments, as well as through the generation of protein crosslinking and formation of protein-quinone adducts. These modifications agree with the reduction of Lys and Arg residues, indicating that concentrations of PEE from *R. cucullatum* should be carefully chosen to achieve antiglycating effects, without affecting the nutritional quality of the proteins.

## Figures and Tables

**Table 1:** Effect of polyphenolic enriched extracts (PEE), anthocyanin fraction (AF) and co-pigment fraction (CF) from *Ribes cucullatum* on the MDA content generated after lipid peroxidation of turkey meat under simulated gastric digestion conditions. Different letters (a, b) in the same column show significant differences compared with *R. cucullatum* PEE, according to Tukey’s test (*p* < 0.05).

Sample	IC_50_ (µg/mL)
*R. cucullatum* PEE	63.7 ± 6.3 ^a^
*R. cucullatum* PEE 60 °C *	88.1 ± 2.5 ^b^
*R. cucullatum* CF	80.2 ± 3.6 ^a^
*R. cucullatum* AF	88.1 ± 5.8 ^b^
Aminoguanidine **	329.5 ± 3.8 ^b^

* Samples incubated at 60 °C during 2 h under an atmosphere of 20% O_2_. ** Reference compound.

**Table 2:** HPLC analysis of ultrafiltered samples containing the main polyphenols present in PEE from *R. cucullatum* (5 mg/mL) exposed to 60 °C during two hours in the presence of sarcoplasmic proteins (3 mg/mL) from chicken.

Compound	PEE + Protein at 4 °C (µg/g)	PEE + Protein at 60 °C (µg/g)
Delphinidin-3-glucoside	52.8 ± 0.5	20.3 ± 0.2 ^a^
Delphinidin-3-rutinoside	61.5 ± 0.1	21.4 ± 0.2 ^a^
Cyanidin-3-glucoside	54.7 ± 0.7	23.0 ± 0.4 ^a^
Cyanidin-3-rutinoside	158.9 ± 0.9	62.9 ± 0.5 ^a^
Chlorogenic acid	1090.5 ± 2.8	782.0 ± 0.1 ^a^

^a^ indicates statistically significant differences (*p* < 0.05) when contrasting the amount of a phenolic compound in PEE samples incubated at 60 °C with the same phenolic in PEE samples incubated at 4 °C.

## Data Availability

Data is contained within the article.
